# Reply: “Refined Balloon Pulmonary Angioplasty” May Need to Be Refined

**DOI:** 10.1016/j.jacadv.2023.100549

**Published:** 2023-08-09

**Authors:** Riyaz Bashir, Vladimir Lakhter, Paul Forfia, William Auger

Thanks to Dr Magod and colleagues for their interest in our study^1^ of refined balloon pulmonary angioplasty (BPA), which reported the data from the inception of our BPA program. Refined BPA, as defined by Mizoguchi et al,^2^ refers to the initial use of smaller balloons followed by larger balloons in follow-up sessions. This conclusion was based on intravascular ultrasound studies rather than pressure wire. Inami et al^3^ later described the pressure-wire-guided BPA and its role in reducing the risk of reperfusion pulmonary edema and lung injury. In our early experience, we wanted to take all possible precautions to improve the safety of this procedure; therefore, we used both the pressure wire guidance and smaller balloons in the initial sessions. Pressure wire use allowed us to identify a hemodynamically significant lesion with high confidence and provided us with pathophysiologic insights while potentially enhancing safety. For the sake of transparency, we did notice the challenges of using a pressure wire during BPA, as pointed out by Dr Magod and colleagues. We addressed these challenges by the following: 1) we used a 300 cm pressure wire with a wireless pressure transducer, which allows better torqueability of the wire tip than a 180 cm pressure wire; 2) use of an angioplasty balloon to support and protect the wire tip, which increases the wire tip's durability; and 3) frequent use of 2 wires in the parent vessel, one to maintain the guide position and the other to cross the lesion. With these technical modifications, its use does not increase the procedure time or change the number of sessions or vessels treated. In fact, we use the same wire and balloon in other lesions, moving from one branch to another without needing multiple exchanges. Since 40% to 50% of the lesions are not crossable by a pressure wire, we agree that routine use of a pressure wire may not be necessary in all BPA procedures, but it does provide us added value in the cases where this approach is utilized. However, we agree with our colleagues that rigorous studies are required to establish optimal BPA techniques to address effectiveness and efficacy and minimize complications.
